# THR0921, a novel peroxisome proliferator-activated receptor gamma agonist, reduces the severity of collagen-induced arthritis

**DOI:** 10.1186/ar1856

**Published:** 2005-11-17

**Authors:** Tetsuya Tomita, Yoshimi Kakiuchi, Philip S Tsao

**Affiliations:** 1 Falk Cardiovascular Research Center, Stanford University School of Medicine, 300 Pasteur Drive, Stanford, CA 94305, USA

## Abstract

THR0921 is a novel peroxisome proliferator-activated receptor gamma (PPARγ) agonist with potent anti-diabetic properties. Because of the proposed role of PPARγ in inflammation, we investigated the potential of orally active THR0921 to inhibit the pathogenesis of collagen-induced arthritis (CIA). CIA was induced in DBA/1J mice by the injection of bovine type II collagen in complete Freund's adjuvant on days 0 and 21. Mice were treated with THR0921 (50 mg/kg/day) starting on the day of the booster injection and throughout the remaining study period. Both clinical disease activity scores as well as histological scores of joint destruction were significantly reduced in mice treated with THR0921 compared to untreated mice. Proliferation of isolated spleen cells, as well as circulating levels of IgG antibody to type II collagen, was decreased by THR0921. Moreover, spleen cell production of IFN-γ, tumor necrosis factor (TNF)-α and IL-1β in response to exposure to lipopolysaccharide or type II collagen was reduced by *in vivo *treatment with THR0921. Steady state mRNA levels of TNF-α, IL-1β, monocyte chemotactic protein-1 and receptor activator of nuclear factor κB ligand (RANKL) in isolated joints were all decreased in mice treated with THR0921. Finally, THR0921 inhibited osteoclast differentiation of bone marrow-derived cells stimulated with macrophage colony-stimulating factor and RANKL. In conclusion, THR0921 attenuates collagen-induced arthritis in part by reducing the immune response. As such, PPARγ may be an important therapeutic target for rheumatoid arthritis.

## Introduction

Rheumatoid arthritis (RA) is characterized by a chronic Th-1 associated inflammatory process resulting in systemic immunological abnormalities and progressive joint destruction. Although the precise etiology and underlying mechanisms of RA remain to be elucidated, several mediators, and thus potential therapeutic targets, have emerged. Proinflammatory cytokines such as tumor necrosis factor (TNF)-α and IL-1β play a key role affecting the imbalance between Th1 and Th2 cells, as well as the activation of other cytokines, chemokines and proteinases [1-5]. In addition, recent evidence indicates that osteoclasts may participate in joint destruction in both animal models of RA and human disease [6,7]. Receptor activator of nuclear factor κB ligand (RANKL) has been reported to be a potent regulator of osteoclast differentiation and activation [8-11]. Recent biological therapies against TNF-α and IL-1β have demonstrated promising effects protecting against progressive joint destruction [12-15]. The clinical use of these therapies has been limited, however, due to several issues, including safety and cost of treatment.

Peroxisome proliferator-activated receptor (PPAR)γ is a member of the ligand-activated transcription factors in the nuclear receptor superfamily and was initially characterized as a key regulator of adipocyte differentiation and lipid metabolism [16,17]. Recent studies have demonstrated that PPARγ agonists prove to have anti-inflammatory and immunomodulatory therapeutic effects [2]. Indeed, the natural PPARγ ligand 15-deoxy-Delta (12,14)-prostaglandin J_2 _as well as synthetic PPARγ agonists such as rosiglitazone and troglitazone have been shown to ameliorate arthritis in rodent models [18,19]. However, the mechanisms for the beneficial effects of PPARγ agonists in arthritis have not been fully delineated. THR0921 is an orally active PPARγ agonist with potent anti-diabetic effects. Its main structural components consist of a thiazolidinedione (TZD) ring covalently linked to a polyphenolic backbone, based on compounds derived from the bark of plants of the *Pterocarpus *genus. Plant extracts from this origin have long been used in the Indian Ayurvedic system of medicine [20,21]. Given the potential role of PPARγ in inflammatory processes, we hypothesized that THR0921 may have protective effects in a murine model of RA. Collagen-induced arthritis (CIA) is a widely used experimental polyarthritis model that has many common histopathological features with human RA. Using this model, we observed marked therapeutic effects of THR0921 upon joint destruction. In addition, we demonstrate that the benefit of THR0921 is due, in part, to inhibitory effects on the production of proinflammatory cytokines, T-cell proliferation, and osteoclastogenesis.

## Materials and methods

### Mice

Female DBA/1J mice (six weeks old) were obtained from the Jackson Laboratory (Bar Harbor, ME, USA). All mice were maintained in a room equipped with an air-filtering system, and the cages and water were sterilized. All procedures performed were in compliance with the Animal Welfare Act and US Department of Agriculture regulations and were approved by Stanford University Animal Care and Use Committee.

### Collagen-induced arthritis

CIA was induced as previously reported [22]. Briefly, six-week old female DBA/1J mice were immunized at the base of the tail with 200 μg of bovine type II collagen (CII) dissolved in 100 μl of 0.05 M acetic acid and mixed with an equal volume (100 μl) of complete Freund's adjuvant (Chondrex Inc., Redmond, WA, USA). On day 21, the mice received a booster injection in the tail of the same volume.

### Experimental protocol

Mice were fed normal chow diet throughout the experimental period. Starting on day 21, a subset of animals was treated with THR0921 (50 mg/kg/day; a generous gift from Dr Coleman Gross, Theracos, Sunnyvale, CA, USA) mixed in with the chow. Animals were sacrificed on day 42 by anesthesia with 2, 2, 2-tribromoethanol and cervical dislocation. Serum samples were collected by intracardiac puncture while tissues were isolated as outlined below.

### Evaluation of development of arthritis

Disease activity of the CIA was assessed every other day between days 21 and 42 by two blinded observers using a three-point scale for each paw: 0, normal joint; 1, slight inflammation and redness; 2, severe erythema and swelling affecting the entire paw, with inhibition of use; and 3, deformed paw or joint, with ankylosis, joint rigidity, and loss of function. The total score for clinical disease activity was based on all four paws, with a maximum score of 12 for each mouse [23].

### Histological studies

Hind paws were harvested from all mice on day 42 and fixed in 4% paraformaldehyde, decalcified with EDTA, and embedded in paraffin; 4 μm sections were prepared. The extent of arthritis was assessed on sections stained with hematoxylin and eosin as previously described [24], using the following scale: 0, normal synovium; 1, synovial membrane hypertrophy and cell infiltrates; 2, pannus and cartilage erosions, 3, major erosions of cartilage and subchondral bone; and 4, loss of joint integrity and ankylosis. The assessment was performed by two independent investigators who were blinded to the identity of the specimens, and the average of the two scores was used.

### Proliferation of spleen cells

*In vitro *proliferation of spleen cells was examined using 3-[4,5-dimethylthiazol-2-yl]-2,5-diphenyltetrazolium bromide (MTT) assay as previously described [25]. Spleens were removed on day 42. Red blood cells were removed by treatment with 0.16 M Tris-NH_4_Cl solution and cell suspensions (5 × 10^6 ^cells/well in a flat-bottom, 96 well plate) were cultured for 72 h at 37°C in 5% CO_2 _in RPMI medium containing 50 μg/ml heat-denatured bovine CII (heated for 10 minutes at 80°C) or 1 μg/ml phytohemagglutinin (PHA). On the day of the assay, MTT (0.5 mg/ml) was added to the medium in each well, and plates were returned to the incubator for 1 h. Plates were centrifuged (500 × *g* for 10 minutes). Supernatants were removed, and 100 μl of dimethyl sulphoxide were added. The plates were agitated in the dark for 10 minutes to dissolve the MTT formazan crystals. The absorbance of the samples was then recorded at 570 nm with background subtracted at 630 nm. Three wells were analyzed for each condition. Data are presented as the percentage of the cells cultured with medium alone. The results are displayed as the mean ± standard error of the mean (SEM) of three separate experiments.

### Enzyme-linked immunosorbant assay quantification of auto-antibody production

Serum levels of anti-mouse CII IgG were assayed using a mouse IgG anti-CII antibody (Ab) assay enzyme-linked immunosorbent assay (ELISA) kit (Chondrex Inc.) on samples derived on day 42. A standard curve was produced using an anti-CII Ab provided with the ELISA kit.

### Cytokine production by cultured spleen cells

Spleens obtained on day 42 were used for measurements of *in vitro *cytokine production. After removal of red blood cells by treatment with 0.16 M Tris-NH_4_Cl solution, suspensions of spleen cells (2 × 10^6 ^cells/well) were incubated for 48 h at 37°C and 5% CO_2 _in supplemented RPMI medium alone, with 50 μg/ml heat-denatured bovine CII, or with 5 μg/ml lipopolysaccharide (LPS) from *Escherichia coli *0111:B4 (Sigma, St Louis, MO, USA). After a 48 h incubation, the supernatants were collected and TNF-α, IL-1β, IL-4, IL-10 and IFN-γ levels were measured using commercially available sandwich ELISAs (Quantikine Mouse ELISA kit, R&D Systems Inc., Minneapolis, MN, USA) according to the manufacturer's protocol.

### Quantitative real-time PCR of joint samples

Total RNA was extracted from hind paws using TRIzol (Life Technologies, Gaithersburg, MD, USA) and RNAeasy kit (Qiagen, Valencia, CA, USA) according to the manufacturers' instructions. Gene expression was determined by analysis of RNA derived from single hind paws of five representative animals from each experimental group. After DNase treatment, cDNA was synthesized from 5 mg of total RNA using MMLV reverse transcriptase (SuperScript II kit, Invitrogen, Carlsbad, CA, USA). Amplification was carried out in triplicate at 50°C for 2 minutes and 95°C for 10 minutes followed by 40 cycles of 95°C for 15 s and 60°C for 1 minute. A threshold cycle (C_T _value) was obtained from each amplification curve using software (Applied Biosystems). Data were expressed as fold changes relative to healthy donor controls using the ΔΔC_T _method as described in the manufacturer's guidelines (Applied Biosystems). A ΔC_T _value was first calculated by subtracting the C_T _value for 18S ribosomal RNA from the C_T _value for each sample. A ΔΔC_T _value was then calculated by subtracting the ΔC_T _value of the control from each group. Fold changes compared with the control were then determined by raising 2 to the ΔΔC_T _power. The primer pairs used in this study were as follows: for TNF-α, forward 5'-GCCTCTTCTCATTCCTGCTT-3', reverse 5'-CACTTGGTGGTTTGCTACGA-3'; for IL-1β, forward 5'-CCCAAGCAATACCCAAAGAA-3', reverse 5'-CATCAGAGGCAAGGAGGAAA-3'; for monocyte chemoattractant protein-1 (MCP-1), forward 5'-AGCCAGATGCAGTTAACGC-3', reverse 5'-CTGATCTCATTTGGTTCGGA-3'; for RANKL, forward 5'-GCTCCGAGCTGGTGAAGAAAT-3', reverse 5'-CCCAAAGTACGTCGCATCTTG-3'.

### Osteoclast differentiation assay

To investigate the effect of THR0921 on osteoclast formation, osteoclast differentiation induced by macrophage colony-stimulating factor (M-CSF, Pepro Tech EC, London, UK) and soluble RANKL (sRANKL, Pepro Tech EC) was monitored using a modified method reported previously [26]. Bone marrow cells were prepared from the femur and tibia of 6-week-old DBA1/J mice and incubated in tissue culture dishes (100 mm dishes) at 37°C in 5% CO_2 _in the presence of recombinant mouse M-CSF (100 ng/ml). After 24 h in culture, the non-adherent cells were collected. The cells were cultured in a 2-well Lab-Tek™ chamber slide (Nalge Nunc International, Rochester, NY, USA) for 7 days with or without THR0921 (10, 50, and 100 nM) in the presence of RANKL (100 ng/ml), M-CSF (20 ng/ml), and activin A (10 ng/ml) in Dulbecco's modified Eagle's medium containing 10% v/v heat-inactivated fetal bovine serum at 37°C in 5% CO_2_. Cells were fixed and stained for tartrate-resistant acid phosphatase (TRAP; a marker enzyme of osteoclasts) using a TRAP staining kit (Hokudo, Sapporo, Japan) [27]. TRAP-positive multinucleated cells containing more than three nuclei were counted under microscopic examination (40× magnification) from four randomly selected fields in each chamber as previously described [28].

### Statistical analysis

The results are expressed as the mean ± SEM. Clinical scores were analyzed by repeated measures analysis of variance with the Sheffe *post-hoc *test. The Mann-Whitney *U* test was used for all other statistical analyses. A *p *value of less than 0.05 was considered significant.

## Results

### Effect of treatment with THR0921 in mice with collagen-induced arthritis

**Figure 1:**
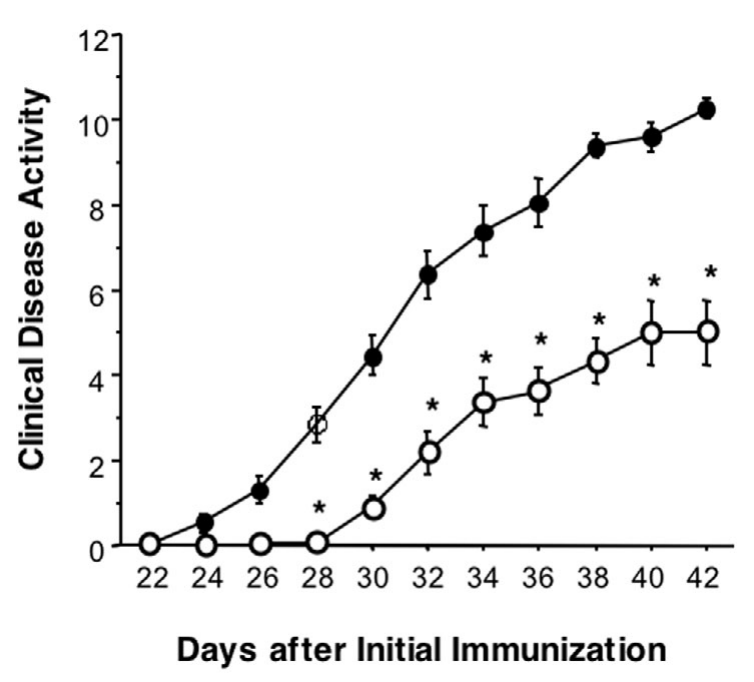
Clinical disease activity of collagen-induced arthritis (CIA). DBA/1J mice were immunized with type II collagen and complete Freund's adjuvant (CFA) on days 0 and 21. The mice were then supplemented with THR0921 (50 mg/kg; open circles) or vehicle (closed circles) for 3 weeks. The clinical disease activity of CIA was determined every other day using a three-point scale for each paw. Data are expressed as mean ± standard error of the mean (n = 10/group; asterisks indicate *p *< 0.001 versus untreated-CIA mice).

**Figure 2:**
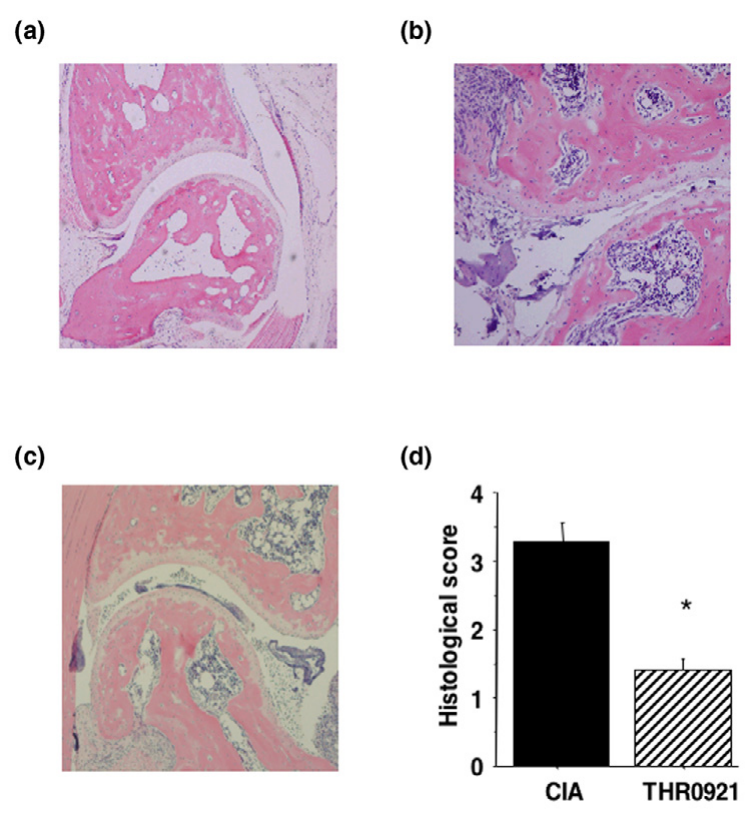
Histological analysis of the hindpaws. **(a) **Representative haematoxylin and eosin stained section from a control mouse shows normal cartilage and absence of infiltrate in the synovium. **(b) **Section from mouse with collagen-induced arthritis (CIA) indicates marked infiltration of leukocytes along with disruption and loss of articular cartilage. **(c) **Section from THR0921 treated CIA mouse showing nearly intact articular cartilage and subchondral bone, and less synovial hyperplasia. **(d) **Mean ± standard error of the mean of histological scores (n = 10/group; asterisks indicate *p *< 0.001).

The incidence of onset of arthritis was 100% in untreated and THR0921-treated CIA groups. Repeated measure analysis of variance demonstrated that THR0921 delayed the onset of arthritis as well as significantly reducing the clinical disease activity score during the course of the experiment compared with CIA mice (Figure 1). From day 28 on, the clinical disease activity score significantly diverged (*p *< 0.001), resulting in a score of 10.3 ± 0.3 and 4.7 ± 2.3 on day 42 in the CIA and the THR0921-treated groups, respectively. The histology of ankle joints from CIA mice showed severe proliferation of synovium with significant inflammatory cell infiltration, pannus invasion, cartilage damage and bone resorption (Figure 2b) compared with normal mice (Figure 2a), while joints from THR0921-treated mice showed mild synovial hyperplasia with less inflammatory cell infiltration, no pannus formation, and little cartilage and bone damage (Figure 2c). The average histological score on day 42 in CIA and THR0921 mice was 3.3 ± 0.8231 and 4 ± 0.516, respectively (*p *= 0.0005; Figure 2d).

### Decreased spleen cell proliferation after treatment with THR0921

**Figure 3:**
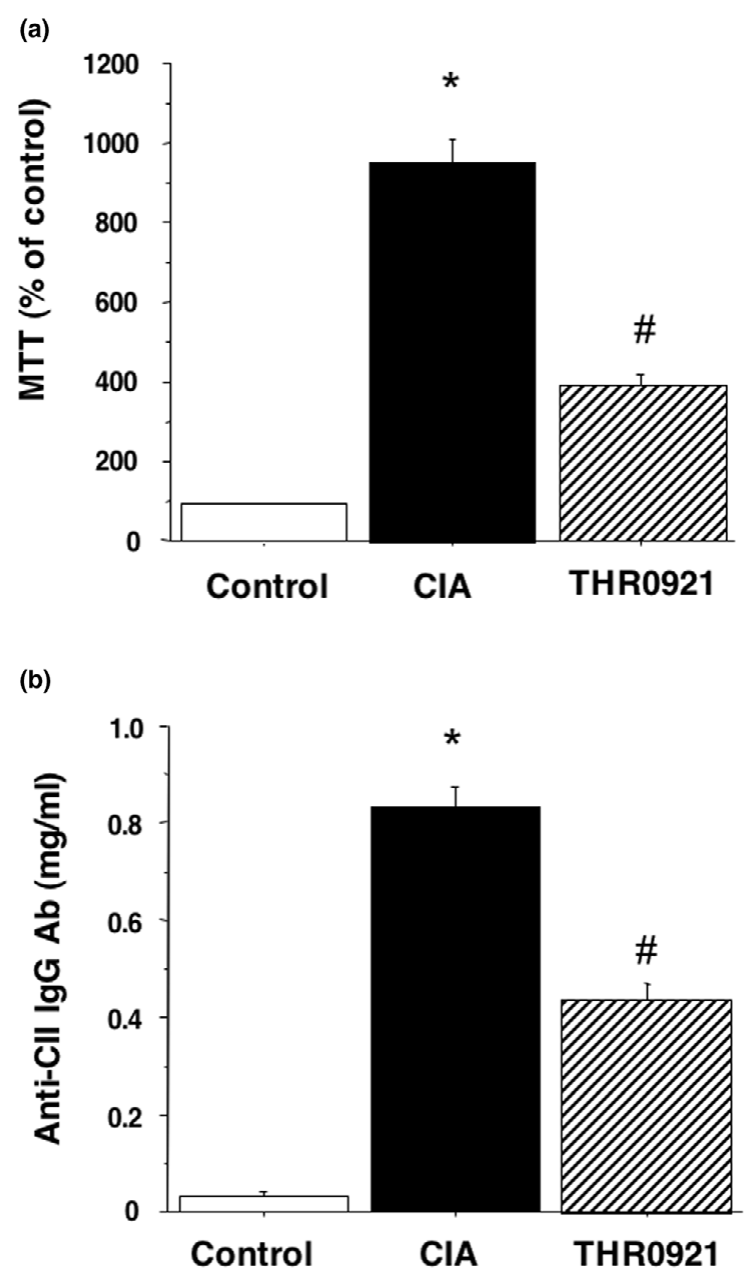
Effects on spleen cell proliferation and CII Ab production. **(a) **Spleen cell proliferation. Spleen cells were isolated from mice on day 42, stimulated with type II collagen (CII) (50 μg/ml) and cultured for 72 h. Cell number was measured by a 3-[4,5-dimethylthiazol-2-yl]-2,5-diphenyltetrazolium bromide (MTT) assay and expressed as percentage of cells derived from control animals. Histograms represent mean ± standard error of the mean (n = 5/group; the asterisk indicates *p *< 0.001 versus control; the hash symbol indicates *p *< 0.01 versus collagen-induced arthritis (CIA) mice without treatment. **(b) **Serum anti-CII antibody (Ab) levels. Ab concentrations in the serum (day 42) were measured by enzyme-linked immunosorbant assay. Histograms represent the mean ± standard error of the mean (n = 10/group; the asterisk indicates *p *< 0.001 versus control; the hash symbol indicates *p *< 0.01 versus CIA mice without treatment).

To determine whether treatment with THR0921 *in vivo *is associated with a cell-mediated immunity to collagen, *in vitro *spleen cell proliferation was measured. There was no significant difference in the values for phytohemagglutinin-stimulated proliferation in spleen cells obtained from healthy controls, CIA and THR0921 mice (data not shown). On the other hand, there was a significant reduction in CII-stimulated proliferation in spleen cells obtained from THR0921-treated mice compared with CIA mice without treatment (*p *< 0.001; Figure 3a). However, CII-stimulated proliferation of spleen cells derived from THR0921 mice was not completely reduced to the level of that from healthy DBA control mice.

To determine whether treatment with THR0921 *in vivo *is associated with a reduction of CII Ab production, serum was collected on day 42 and analyzed for the anti-CII IgG Ab by an ELISA assay. The anti-CII IgG Ab levels in the serum of THR0921-treated CIA mice were significantly lower than those in untreated CIA mice (*p *= 0.0002; Figure 3b).

**Figure 4:**
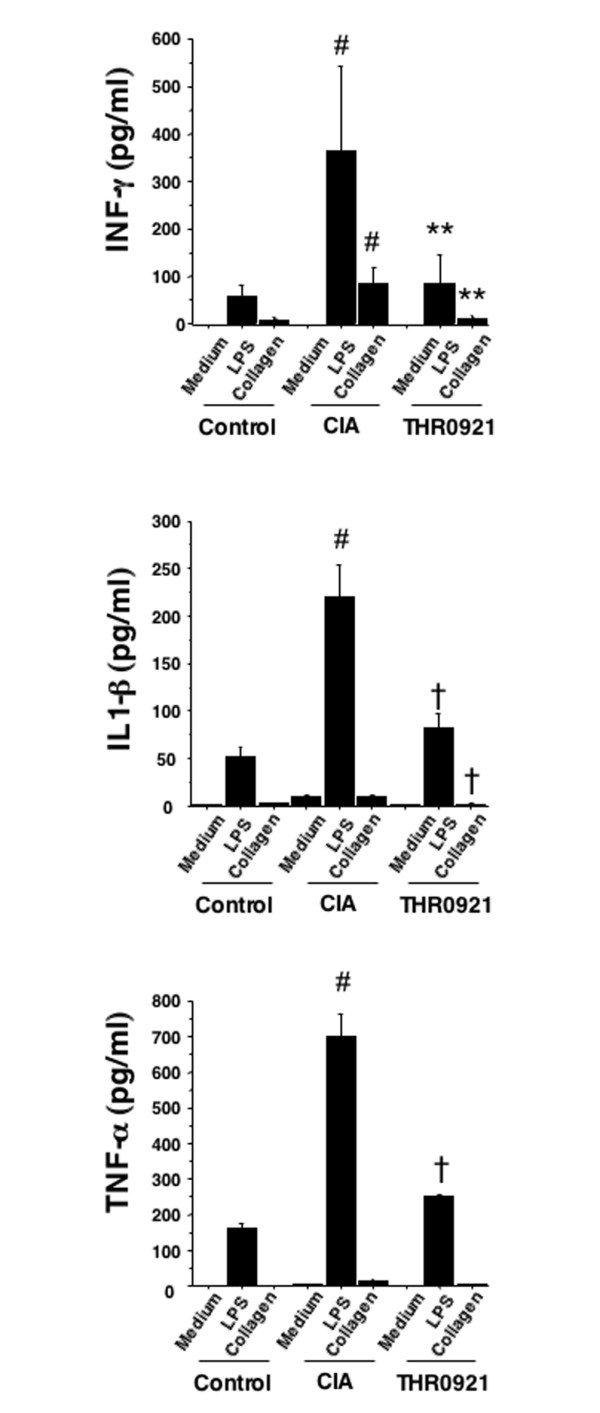
Cytokine production by spleen cells. Spleens were obtained from control DBA/1J (Control) collagen-induced arthritis (CIA) mice, and THR0921 treated mice at day 42 and single cell suspensions were cultured with medium alone, 50 μg/ml type II collagen, or 5 mg/ml lipopolysaccharide (LPS) for 48 h. The levels of INF-γ, IL-1β, and tumor necrosis factor (TNF)-α were measured in supernatants using a specific enzyme-linked immunosorbant assay. Histograms represent the mean ± standard error of the mean (n = 10/group; the hash symbol indicates p < 0.01 from control; the dagger symbol indicates p < 0.05 versus CIA; asterisks indicate *p *< 0.01 versus CIA).

As inflammatory cytokines are thought to play a critical role in the pathogenesis of RA, we monitored the production of the proinfammatory cytokines TNF-α, IL-1β, and INF-γ as well as anti-inflammatory IL-4 and IL-10 in the spleen cell supernatants by ELISA. *In vivo *treatment with THR0921 resulted in reduced production of TNF-α, IL-1β, and INF-γ by spleen cells cultured for 48 h with either LPS or CII compared with cells from vehicle-treated CIA mice (Figure 4). On the other hand, no difference was observed in elaborated levels of IL-4 and IL-10 between spleen cells derived from any of the groups (data not shown).

### Cytokine expression in ankle joints

**Figure 5:**
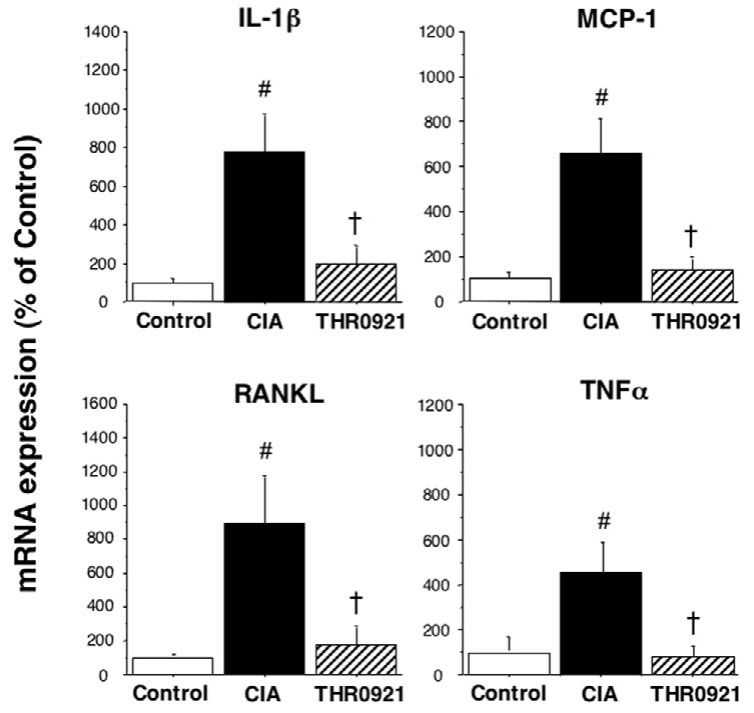
Steady state mRNA levels of the inflammatory cytokines IL-1β, monocyte chemotactic protein (MCP)-1, receptor activator of nuclear factor κB ligand (RANKL) and tumor necrosis factor (TNF)-α. Joints were isolated from mice on day 42, and total RNA was extracted. Cytokine gene expression was determined by real time PCR using the ΔΔCT method and is expressed as a percent of control (n = 5/group; the hash symbol indicates *p *< 0.01 from control; the dagger symbol indicates *p *< 0.05 versus untreated collagen-induced arthritis (CIA)).

To investigate the effect of oral administration of THR0921 at the site of joint destruction, the expression levels of TNF-α, IL-1β, MCP-1 and RANKL mRNA were examined by real-time RT-PCR analysis. As expected, expression of each of these cytokines was significantly increased with CIA (*p *< 0.001); treatment with THR0921 reduced cytokine levels nearly to control levels (*p *< 0.01; Figure 5).

### Inhibitory effect of THR0921 on osteoclast differentiation of bone marrow cells induced by M-CSF and RANKL *in vitro*

**Figure 6:**
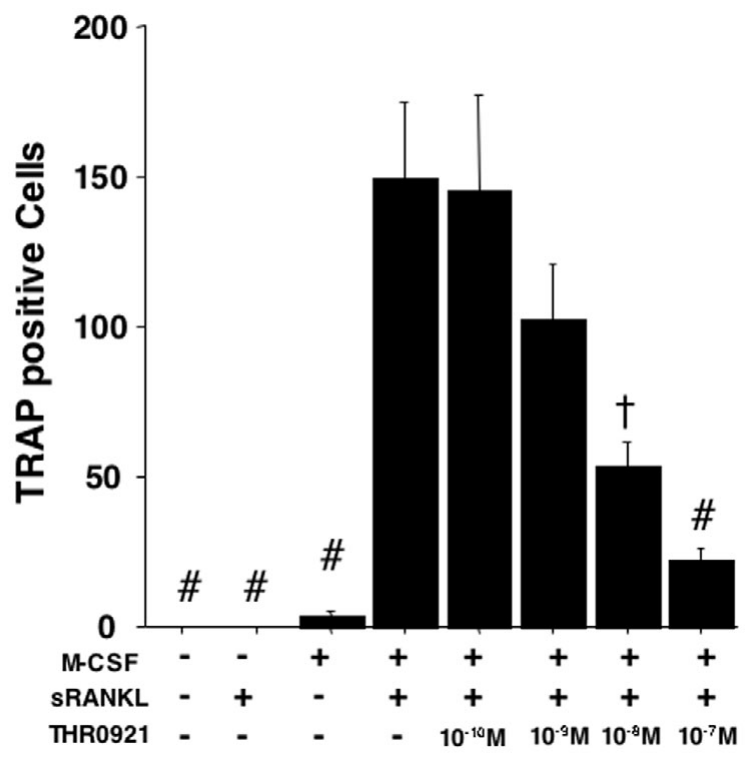
Effect of THR0921 on osteoclast differentiation. Mouse bone marrow cells were incubated for 8 days in the presence or absence of macrophage colony-stimulating factor (M-CSF; 10 ng/ml), soluble receptor activator of nuclear factor κB ligand (sRANKL; 30 ng/ml), and THR0921 at the concentrations shown. After incubation, cells were stained with the tartrate-resistant acid phosphatase (TRAP) staining kit and enumerated under 40× magnification. The mean ± standard error of the mean of TRAP-positive multinuclear cells containing three or more nuclei from four randomly selected fields is shown. The hash symbol indicates *p *< 0.01; the dagger symbol indicates *p *< 0.05 versus cells stimulated with M-CSF + sRANKL without THR0921.

As osteoclasts play an important role in bone destruction of inflamed joints, we investigated the effect of THR0921 on osteoclast differentiation of bone marrow-derived cells. M-CSF and sRANKL induced differentiation of bone marrow cells derived from normal and arthritic mice was monitored by TRAP-positive multinucleated cell formation; this effect was dose-dependently reduced with THR0921 treatment. Similar effects were observed when osteoclastogenesis was stimulated with RANKL (*p *< 0.001 for 10^-7 ^M, and 10^-8 ^M of THR0921 compared with no THR0921; Figure 6).

## Discussion

In this study, oral administration of THR0921 resulted in marked reduction in inflammatory severity as well as histological degree of CIA without any obvious adverse effects. Although the prophylactic study design limits immediate clinical translation, the results indicate an important regulatory role for PPARγ and suggest further study is warranted.

Although the exact pathogenesis of RA is partly unknown, it is clear that the disease process is characterized by infiltration of leukocytes and erosion of cartilage and bone. Recent studies have indicated that many of the cells involved with RA, namely mononuclear leukocytes [29], articular cartilage and chondrocytes, each express PPARγ receptors [30]. Thus, there are several potential mechanisms by which THR0921 can protect against CIA. Previous reports have highlighted the participation of locally produced cytokines in the inflammation as well as tissue destruction, even in the early phases of experimental arthritis [31-33]. We observed that oral administration of THR0921 resulted in reduced concentrations of proinflammatory cytokines at both the local and systemic levels. Of particular interest, THR0921 suppressed levels of MCP-1, a chemokine responsible for monocyte recruitment and activation. Consistent with these results are previous observations indicating that PPARγ agonists can inhibit activation of monocyte/macrophages [34]. As macrophages initiate the inflammatory process and are the source of many cytokines responsible for cartilage and bone loss, this may be a primary mechanism by which THR0921 provides clinical benefit. The effect of THR0921 and other PPARγ ligands may be due to their ability to inhibit the activity of key transcription factors such as activator protein-1 (AP-1) and nuclear factor κB [35,36]. As such, targeted therapy against nuclear factor κB activation has proven successful in experimental arthritis models [35,36].

In addition to stimulated macrophages in the affected joints, the pathogenesis of CIA is also dependent upon T-cell activation [37]. Prior studies have shown that T-cells are involved with the initiation of synovial hyperplasia, an early step leading to bone destruction. In addition, T-cell infiltrate is directly correlated to severity of arthritis. As such, our observation that THR0921 reduced CII-induced spleen cell proliferation may indicate a major mode of protection in this model. Indeed, as a PPARγ agonist, THR0921 may be expected to alter T-cell function. Clark *et al*. [38] demonstrated that PPARγ ligands significantly blocked the proliferative response of both T-cell clones and freshly isolated splenocytes. In addition, we observed reduced circulating levels of CII Ab in THR0921 treated animals, thereby providing less of a stimulus for an immune response [39,40]. A potential drawback of the current findings, however, is that we have not clearly demonstrated a change in spleen cell population (for example, T-cell content) by THR0921 treatment. Thus, we cannot conclude that the protective effect was due to reduced T-cell number.

Less controversial, however, are the observed effects upon spleen cell activation. Chronic treatment with THR0921 resulted in reduced production of TNF-α, IL-1β, and INF-γ from isolated spleen cells induced by LPS. As the involvement of each of these cytokines in the pathogenesis of arthritis is well known [41-43], this would clearly have a major impact on disease development. On the other hand, we did not observe any effects upon the levels of the anti-inflammatory cytokines IL-4 and IL-10. These results suggest that the major effect of THR0921 is to suppress proinflammatory mechanisms as opposed to activating counter-regulatory gene transcription.

Suppression of osteoclast formation and activation has emerged as a pivotal therapeutic strategy used against joint destruction in arthritis. In the current study, THR0921 clearly inhibited the differentiation and maturation of osteoclast-like cells induced by sRANKL and M-CSF in the mouse bone marrow culture assay in a dose dependent manner. Of note, THR0921 almost completely inhibited the osteoclastogenesis *in vitro *at a concentration equivalent to serum levels achieved in mice (for example, 10^-6 ^M; data not shown). These data suggest that THR0921 may directly act on the lineage of macrophages/monocytes that differentiate into bone-resorbing osteoclasts. The use of sRANKL as a stimulus is supported by recent studies implicating it as an essential regulator of osteoclast recruitment and differentiation, as well as its crucial role in rheumatoid joint destruction [9]. It is, therefore, intriguing that THR0921 had the additional effect of reducing local expression of RANKL in affected joints.

THR0921 has a spectrum of activity that differs from other commercially available thiazolidinediones (TZDs). It is important to note that the anti-inflammatory effects observed with THR0921 in the current study were achieved with a dose that was 10 times less than that used for troglitazone in a similar model of arthritis [2]. This is particularly surprising because THR0921 is a weak activator of PPARγ compared to rosiglitazone and has less adipogenic activity. This may also be advantageous, however, when considering other side effects of TZD treatment. For example, THR0921 is expected to produce less weight gain than current commercially available PPARγ activators. Moreover, THR0921 would be expected to produce less edema, an important consideration in arthritis therapy. Furthermore, the oral formulation of THR0921 would provide greater safety and convenience over current anti-cytokine therapies that are delivered by injection.

## Conclusion

We demonstrate that oral administration of THR0921 potently attenuates the development of progressive joint destruction in mice with CIA. This effect is due, in part, to its ability to inhibit T-cell proliferation, reduce proinflammatory cytokine production, and suppress osteoclastic bone resorption. The findings in this study suggest that THR0921 may be developed into a potential therapeutic agent for arthritis.

## Abbreviations

Ab = antibody; CIA = collagen-induced arthritis; CII = type II collagen; ELISA = enzyme-linked immunosorbant assay; IL = interleukin; INF = interferon; LPS = lipopolysaccharide; MCP = monocyte chemotactic protein; M-CSF = macrophage colony-stimulating factor; MTT = 3-[4,5-dimethylthiazol-2-yl]-2,5-diphenyltetrazolium bromide; PPAR = peroxisome proliferator-activated receptor; RA = rheumatoid arthritis; RANKL = receptor activator of nuclear factor κB ligand; RT-PCR = reverse transcriptase polymerase chain reaction; SEM = standard error of the mean; sRANKL = soluble RANKL; TNF = tumor necrosis factor; TRAP = tartrate-resistant acid phosphatase; TZD = thiazolidinedione.

## Competing interests

The authors declare that they have no competing interests.

## Authors' contributions

TT conceived, designed, and performed the majority of the described studies and was responsible for initial versions of this manuscript. YK designed and performed many of the inflammatory and gene expression assays included in this manuscript. PST helped design and oversaw the completion of the studies as well as the writing of this manuscript.
